# Prenatal Exposure to Mercury, Manganese, and Lead and Adverse Birth Outcomes in Suriname: A Population-Based Birth Cohort Study

**DOI:** 10.3390/toxics10080464

**Published:** 2022-08-11

**Authors:** Vinoj H. Sewberath Misser, Ashna D. Hindori-Mohangoo, Arti Shankar, Jeffrey K. Wickliffe, Maureen Y. Lichtveld, Dennis R. A. Mans

**Affiliations:** 1 Department of Pharmacology, Faculty of Medical Sciences, Anton de Kom University of Suriname, Kernkampweg 5-7, Paramaribo, Suriname; 2 Department of Environmental Health Sciences, School of Public Health and Tropical Medicine, Tulane University, New Orleans, LA 70112, USA; 3 Foundation for Perinatal Interventions and Research in Suriname (Perisur), Paramaribo, Suriname; 4 Department of Environmental Health Sciences, School of Public Health, University of Alabama at Birmingham, Birmingham, AL 35294, USA; 5 School of Public Health, University of Pittsburgh, Pittsburgh, PA 15260, USA

**Keywords:** mercury, manganese, lead, blood levels, stillbirth, preterm birth, low birth weight, low Apgar score

## Abstract

Globally, adverse birth outcomes are increasingly linked to prenatal exposure to environmental contaminants, such as mercury, manganese, and lead. This study aims to assess an association between prenatal exposure to mercury, manganese, and lead and the occurrence of adverse birth outcomes in 380 pregnant women in Suriname. The numbers of stillbirths, preterm births, low birth weights, and low Apgar scores were determined, as well as blood levels of mercury, manganese, lead, and relevant covariates. Descriptive statistics were calculated using frequency distributions. The associations between mercury, manganese, and lead blood levels, on the one hand, and adverse birth outcomes, on the other hand, were explored using contingency tables, tested with the χ^2^-test (Fisher’s exact test), and expressed with a *p* value. Multivariate logistic regression models were computed to explore independent associations and expressed as (adjusted) odds ratios (aOR) with 95% confidence intervals (CI). The findings of this study indicate no statistically significant relationship between blood mercury, manganese, or lead levels and stillbirth, preterm birth, low birth weight, and low Apgar score. However, the covariate diabetes mellitus (aOR 5.58, 95% CI (1.38–22.53)) was independently associated with preterm birth and the covariate hypertension (aOR 2.72, 95% CI (1.081–6.86)) with low birth weight. Nevertheless, the observed high proportions of pregnant women with blood levels of mercury, manganese, and lead above the reference levels values of public health concern warrants environmental health research on risk factors for adverse birth outcomes to develop public health policy interventions to protect pregnant Surinamese women and their newborns from potential long-term effects.

## 1. Introduction

Globally, stillbirth, preterm birth, low birth weight, and low Apgar score are among the most common adverse birth outcomes. Each year, roughly 15 million babies are born prematurely [1], about 2 million die before or during delivery [2], approximately 20 million have a low birth weight [3], and a low Apgar score has been related to the death of approximately 4 million babies worldwide [4]. Adverse birth outcomes and their consequences represent a critical public health issue in low- and middle-income countries, in particular, where resources for adequate healthcare are, in general, insufficient [3,5]. For instance, from 2000 to 2019, 98% of stillbirths were registered in low- and middle-income countries [2]; in 2014, more than 60% of preterm births were in South Asia and sub-Saharan Africa [6,7]; and in 2013, nearly 22 million newborns with a low birth weight—or 16% of all babies born globally—were found in Asia and Africa [8].

Several factors have been associated with adverse birth outcomes. Some of these include socio-demographic characteristics of the mother, such as age at the birth of the child, marital status, education, occupation, household income, and nutritional status [9,10,11], and her reproductive and obstetric characteristics, such as age of sexual debut, parity, and history of perinatal death [12,13]. The accessibility and availability of maternal health services and the quality of antenatal care also play a role in the occurrence of adverse birth outcomes [13,14,15]. Other major causes of adverse birth outcomes are exposure to environmental pollutants during pregnancy, such as pesticides and herbicides, polycyclic aromatic hydrocarbons, particulate matter, and toxic heavy metals [16,17,18]. These exposures have also been linked to long-term neurodevelopmental deficits in young children [19,20].

The Republic of Suriname is a South American country that borders the Atlantic Ocean to the north and is located between French Guiana and Guyana. Suriname’s major means of subsistence are gold mining and agriculture, in addition to crude oil drilling, fisheries, forestry, and ecotourism [21]. Together, these economic activities have resulted in a gross domestic income in 2020 of USD 2.88 billion and an average per-capita income in that year of about USD 4900 [22]. This positions Suriname on the World Bank’s list of upper–middle income economies [22].

Regrettably, these developments have been achieved at the expense of severe environmental pollution, massive land degradation, and extensive deforestation [23]. The use of considerable amounts of mercury to amalgamate the gold produced in artisanal gold mining activities [24] has led to the poisoning of streams, rivers, and lakes as well as species of fish for human consumption [25,26]. Consequently, unacceptably high mercury levels (particularly in the form of methylmercury) have been detected in various preferred fish species that are high on the food chain, such as the anyumara or wolf fish *Hoplias aimara* [27,28]. The need to produce increasing amounts of field crops, fruits, and vegetables has resulted in the unregulated and illegal use of banned agricultural pesticides and herbicides that contain, among others, manganese and lead. Manganese is one of the active ingredients of the ethylene bis-dithiocarbamate fungicide mancozeb [29] that is widely used in the large-scale cultivation of bananas and rice [30,31]. Lead is present in glyphosate-based herbicides and has been detected in agricultural products, such as cassava [32,33]. This compound has even been detected in drinking water from the national distribution system [34].

When these heavy metals enter the food chain and are ingested by pregnant women, they increase the risk of adverse birth outcomes. Prenatal exposure to mercury has been associated with reduced placental and fetal growth [35,36], while methylmercury compounds have been related to neurological disorders of the child [35,37]. Manganese exposure during pregnancy predisposes newborns to a lower birth weight [38,39,40], and higher maternal lead concentrations increase the risk of preterm birth [36,41] and overweight or obesity of the child [42].

The hospital-based maternal mortality rate in Suriname over the past thirty years was 127 per 100,000 [43], the stillbirth rate was 16 per 1000 births, and the preterm birth and low birth weight rates were 14% and 15%, respectively [44]. Whether these adverse birth outcomes are attributable to overexposure to mercury, manganese, or lead during pregnancy is not known. Hence, this study aims to assess an association between prenatal exposure to mercury, manganese, and lead and the occurrence of adverse birth outcomes in Suriname. For this purpose, the current study was embedded in the Caribbean Consortium for Research in Environmental and Occupational Health (CCREOH)’s prospective cohort study, in which the blood levels from a sub-cohort of 400 pregnant women from three geographic regions of Suriname were analyzed [45].

The influence of several factors that were not the focus of the current study but may have an impact on adverse birth outcomes have also been assessed. These covariates included medical conditions, such as diabetes mellitus [46,47], hypertension [48], and anemia [44,49,50], as well as maternal age older than 35 years [51,52], multiparity [53,54], alcohol use until at least 3 months before pregnancy [55,56], smoking until at least 3 months before pregnancy [57,58], and the use of prescription medicines such as analgesics [59]. Insight into the exposure patterns to the heavy metals will help design interventions and spark future research on environmental and health policies to ensure the safety of pregnant women and their newborns in Suriname.

## 2. Subjects and Methods

### 2.1. Study Design and Setting

In this study, the possible association of prenatal exposure to mercury, manganese, and lead, on the one hand, and adverse birth outcomes in pregnant women in Suriname, on the other hand, was analyzed. As mentioned before, for the current study, the data analyzed were from the Caribbean Consortium for Research in Environmental and Occupational Health (CCREOH)’s cohort of 1200 pregnant women in 2017. The number of 1200 participants was calculated based on a multiple linear regression model using a coefficient of determination of 0.10 and an R2 differential of 0.02 to have 80% power at a 0.05 level of significance, taking into account the potential loss of participants [45]. In this cohort, the pregnant women were enrolled in a longitudinal epidemiological study that evaluated the effects of environmental contaminants on birth outcomes and the neurological development of children born in Suriname [45].

The current study is based on a subset of 400 participants whose blood metal levels were available and have been analyzed. The CCREOH study has been approved by the Institutional Review Board of the Ministry of Health of Suriname (protocol number VG 023-14) and the Institutional Review Board of Tulane University, New Orleans, LA, USA (protocol number 83 093). Each participant entered the study voluntarily, gave informed consent after the purpose of the study was thoroughly explained, and agreed to donate venous blood.

### 2.2. Collection of Maternal Socio-Demographic and Dietary Data

Enrollment of the participants and collection of maternal socio-demographic and dietary data was conducted by trained recruiters through face-to-face-interviews using questionnaires on encrypted iPads, as described in the CCREOH’s study protocol [46].The following maternal socio-demographics were included this study: age at delivery (16–19 years, 20–34 years, or 35 years and older, according to the reproductive age categories of the World Health Organization [60]); region of residence (urban-coastal, rural-coastal, or rural-interior region of Suriname as distinguished by the Surinamese General Bureau of Statistics [61]); monthly household income ≤ USD 75 (SRD 1500) or >USD 75 (SRD 1500); educational level (none or primary education or secondary or tertiary education); and food consumption.

### 2.3. Collection of Data on Birth Outcomes

Data on birth outcomes of the study participants were retrieved from their birth and medical records at the hospitals and primary healthcare clinics where the delivery took place. Stillbirth was defined as a fetus born with no signs of life from 22+ completed weeks of gestation or a birthweight of 500+ g [62,63,64,65]; preterm birth was defined as a birth between 22 + 0 and 36 + 6 weeks of gestation [66]; a low birthweight was defined as a weight at birth of less than 2.500 g regardless of the gestational age [67]; and a low Apgar score was defined as a score of less than seven at 5 min after birth [68].

### 2.4. Determination of Blood Concentrations of Mercury, Manganese, and Lead

Whole blood samples from the 400 study participants were collected during the first and second trimesters of their pregnancy using trace element vacutainers with potassium EDTA. The blood samples were frozen and shipped to the Wisconsin State Laboratory of Hygiene (WSLH) at the University of Wisconsin–Madison (Madison, WI, USA). The Trace Element Research Group of that institution determined the concentrations of mercury, manganese, and lead in the samples using sector field inductively coupled plasma mass spectrometry.

The detection limits for mercury, manganese, and lead were 0.05 µg/L, 0.07 µg/L, and 0.06 µg/L, respectively. Blood levels of mercury were categorized as <3.5 µg/L and ≥3.5 µg/L, using 3.5 µg/L as the reference level [26,69,70]. Those for manganese were categorized as <13.0 µg/L or ≥13.0 µg/L [71]. Using the reference level 3.5 µg/dL, the blood lead levels of were categorized as <3.5 µg/dL and ≥3.5 µg/dL [72]. The reference level cutoff points are referred to as public health action levels.

### 2.5. Covariates Included in the Study

The covariates considered relevant to the current study included elevated levels of blood glucose (≥6.1 mmol/L, obtained after fasting) [73], raised blood pressure (>120/80 mm Hg) [74], and low hemoglobin levels (anemia) (<6.8 mmol/L) [75], as well as maternal age 35 years and over, multiparity (at least 1 previous live birth), alcohol use until at least 3 months before pregnancy, smoking until at least 3 months before pregnancy, and the use of prescription medicines (at least one medicine prescribed). These data were retrieved from the medical records (based on laboratory results) of the participants and from information obtained by interviewing them.

### 2.6. Statistical Analyses

Descriptive statistics were calculated using frequency distributions. The potential associations between prenatal exposure to mercury, manganese, and lead, on the one hand, and adverse birth outcomes, on the other hand, were explored using contingency tables, tested with the χ^2^-test, and expressed with a *p* value. Fisher’s exact test was performed to adjust for sensitivity. Logistic regression models were computed to explore crude and independent associations of the categorized blood levels of the heavy metals and the covariates with the adverse birth outcomes and expressed as crude and adjusted odds ratios (OR) with 95% confidence intervals (CI). Only variables that were statistically significantly associated at the bivariate level at *p* < 0.10 were included in the multivariate models. In the crude models, each variable was tested individually against the relevant birth outcome. In the adjusted models, the variables were evaluated together against the relevant birth outcome to establish associations between the variables. All analyses were conducted using IBM “Statistical Package for the Social Sciences” (SPSS) version 25.

## 3. Results

### 3.1. Sociodemographic Characteristics

Due to missing data on adverse birth outcomes, 20 participants were excluded from the analyses. The socioeconomic characteristics of the remaining 380 women are presented in Table 1. Their average age was 28.5 ± 6.3 years. The majority (74.7%) were 20–34 years old, 15.3% were 35 years or older, and 10.0% were aged between 16 and 19 years. About two-thirds of the women resided in Suriname’s urban–coastal region, 22.4% in the country’s rural–coastal region, and 11.6% in the rural–interior part. Around 30% had an income of less than USD 75 (SRD 1500), and about 18% had received no or only primary education. Together with rice, leafy vegetables, and bread, fish was among the most consumed items, being part of the diets of more than 95% of the participants.

### 3.2. Adverse Birth Outcomes

A total of 74 of the 380 evaluable women had experienced an adverse birth outcome (Table 1). The most frequent adverse birth outcomes were preterm birth, which occurred in 50 cases (13.3%), and low birthweight, which was seen in 43 cases (11.6%). Stillbirth and low Apgar score were registered in 17 and 19 cases, respectively, corresponding to 4.5% and 5.2%, respectively, of the 380 pregnancies (Table 1).

### 3.3. Blood Concentrations of Mercury, Manganese, and Lead

As shown in Table 1, more than one-third of the 380 women (154 or 40.5%) had blood mercury levels ≥ 3.5 µg/L. However, elevated blood levels of manganese (≥13.0 µg/L) were seen in about a 1.5 times higher number, namely in 243 women or 63.9% (Table 1). On the other hand, 81 or 21.3% of women had blood lead levels above the reference level of 3.5 µg/dL (Table 1). Thus, in the period covered by the study, elevated blood levels of mercury and manganese were seen in about two of five and three of five pregnant women, respectively, while high blood lead levels were seen in roughly one of five of the women.

### 3.4. Covariates

Assessment of the covariates considered relevant to the current study (Table 1), showed that 15 of the 380 participants (10.9%) were diabetics, 55 (32.7%) suffered from hypertension, and more than two-thirds (n = 203; 84.6%)) had anemia. In addition, 58 (15.3%) were 35 years or older, more than two-thirds (n = 265; 70.1%) were multiparous, more than half (n = 173; 57.8%) had used alcohol until at least 3 months before their pregnancy, a minority (n = 18; 4.8%) had smoked until at least 3 months before their pregnancy, and more than half (n = 224; 58.9%) had used prescription medicines (for instance, medicines for blood and blood-forming organs, including anti-anemic drugs, as well as medicines for alimentary tract and metabolism, including drugs for acid-related disorders). When compared to the frequencies of women with heavy metal blood levels that were too high, these frequencies were sufficiently high to suggest that these covariates might impact adverse birth outcomes.

### 3.5. Associations between Prenatal Exposure to Heavy Metals or Covariates and Adverse Birth Outcomes

The results from bivariate associations between the blood levels of the heavy metals or the covariates, on the one hand, and the occurrence of adverse effects, on the other hand, are shown in Table 2. No statistically significant associations were found between the blood levels of mercury or manganese and the occurrence of stillbirth, preterm birth, low birth weight, or low Apgar scores (*p* values > 0.10). Maternal age 35 years and over, underlying anemia, multiparity, and smoking were also not statistically significantly associated with any of the adverse birth outcomes (*p* values > 0.10).

On the other hand, blood concentrations of lead ≥3.5 µg/dL were statistically significantly associated with the occurrence of preterm birth (*p* value of 0.065), though not with that of stillbirth, low birth weight, and low Apgar score (*p* values of 0.220, 0.333, and 0.266, respectively). The presences of diabetes mellitus, alcohol consumption, and the use of prescription medicines were also statistically significantly associated with preterm birth (*p* values of 0.028, 0.034 and 0.006, respectively). The same held true for alcohol consumption, as well as underlying hypertension and the occurrence of low birth weight (*p* values of 0.015, 0.015, respectively). Together, these observations suggest that high lead blood concentrations, as well as the presence of diabetes mellitus, alcohol consumption until at least 3 months before pregnancy, and the use of prescription medicines may represent independent variables for the occurrence of preterm birth, and that underlying hypertension and, the use of alcohol until at least 3 months before pregnancy may represent independent variables for the occurrence of low birth weight.

Following up on these findings, multivariate logistic regression models were conducted to determine whether blood lead levels ≥ 3.5 µg/dL underlying diabetes mellitus or hypertension, pre-pregnancy alcohol use, and prescribed medicine use were independent predictors of preterm birth or low birth weight. As shown in Table 3, in the crude model, women with blood lead levels ≥ 3.5 µg/dL had a 1.88 (95% CI 0.98–3.60) higher risk of having a preterm birth when compared to those with blood lead levels < 3.5 µg/dL. Furthermore, the odds of having a preterm birth were 4.29 (95% CI 1.26–14.62) times higher in diabetic women than non-diabetic women, 2.53 (95% CI 1.28–5.00) times higher in women who had prescribed medicines than in those who had not, and 0.50 (95% CI 0.27–0.93) times lower in those who had used alcohol at least 3 months before pregnancy than in those who had not (Table 3).

Further, women with an elevated blood pressure had 3.16 (95% CI 1.28–7.76) times greater odds of giving birth to a baby with a low birth weight than women with a normal tension, while pre-pregnancy alcohol use was associated with a decreased risk of 0.43 (95% CI 0.22–0.85) when compared to the abstinence of alcohol (Table 4). Thus, the ORs found in the crude models indicate that each of the individual variables (diabetes mellitus, hypertension, prescription medicine use, and alcohol intake at least 3 months before pregnancy) were predictive factors for preterm birth or low birth weight.

Next, the influence of underlying diabetes mellitus, hypertension, prescribed medicine, or alcohol intake on the occurrence of preterm birth or low birth weight was explored in the adjusted models. In these analyses, the aOR for an association between blood lead levels ≥ 3.5 µg/dL and preterm birth decreased from 1.88 (95% CI 0.98–3.60) to 1.38 (95% CI 0.40–4.79), and the association between prescription medicine use, as well as pre-pregnancy alcohol use, and preterm birth changed from 2.53 (95% CI 1.28–5.00) to 2.1 (95% CI 0.43–10.16) and 0.50 (95% CI 0.27–0.93) to 0.36 (95% CI 0.11–1.145), respectively (Table 3). The OR of a preterm birth for women with diabetes mellitus increased from 4.29 (95% CI 1.26–14.62) to 5.58 (95% CI 1.38–22.83) after adjustment (Table 3). Thus, as expected, diabetes mellitus can be considered an independent risk factor for preterm birth. Furthermore, women with high blood pressure had 2.72 (95% CI 1.081–6.86) times higher odds of having a low-birth-weight newborn (Table 4). On the other hand, the OR of 0.43 (95% CI 0.22–0.85) changed to 0.47 (95% CI 0.18–1.23) for low-birth-weight babies when using alcohol at least 3 months before pregnancy (Table 4).

Together, these findings suggest that there was no association between prenatal exposure to mercury, manganese, or lead and adverse birth outcomes, but that the covariates of high blood glucose levels and preterm birth, and high blood pressures and low birth weight, showed to be independently related to the adverse birth outcomes.

## 4. Discussion

In this study, the association of blood levels of mercury, manganese, and lead, on the one hand, and the occurrence of adverse birth outcomes, including still birth, preterm birth, low birth weight, and low Apgar score, on the other hand, was assessed in 380 pregnant Surinamese women. Covariates, such as underlying hypertension, diabetes mellitus, and anemia, as well as multiparity, age over 35 years, alcohol use until at least 3 months before pregnancy, smoking until at least 3 months before pregnancy, and the use of prescription medicines, such as anti-anemic drugs, were also included in the analyses.

The women enrolled in the study were part of a larger group of 1200 women who had given birth and in whom the effects of environmental contaminants on birth outcomes and neurological development had been studied [45]. Their average age of 28.5 ± 6.3 years and the distribution of the age groups (about three-quarters aged between 20 and 34 years), regions of residence (about two-thirds living in Suriname’s urban–coastal region), monthly household income (USD 75 or SRD 1.500 for almost two-thirds), education levels (secondary or tertiary education for more than 80%), and food consumption (bread, rice, fish, and leafy vegetables were the most commonly consumed items by at least 80%) represented a good cross-section of the pregnant Surinamese woman [76,77,78,79].

The frequency of adverse birth outcomes in the current sub-cohort (19.5%) and the frequency distribution of the birth outcomes (13.3% preterm births, 11.6% low birth weight, 4.5% stillbirth, and 4.2% low Apgar score) were also roughly within the same range as those previously mentioned for Suriname [44,80] and for fourteen other lower–middle-income countries in several parts of the world [81].

While investigating this group of pregnant Surinamese women, no statistically significant associations were found between blood levels of mercury, manganese, or lead and the occurrence of stillbirth, preterm birth, low birth weight, or low Apgar score. This was unexpected since blood concentrations of the heavy metals were above public health action levels in 40.5%, 63.9%, and 21.3%of the women, respectively. As mentioned before, these compounds are present in various (banned) agricultural pesticides that are, nevertheless, abundantly used in Suriname [82]. Residues of these substances have been detected in tap water [34] and foods that are among the most consumed by pregnant Surinamese women, such as leafy vegetables [83] and fish [26]. The elevated blood mercury levels also coincided with those found in the hair and blood samples of women and children from interior villages in Suriname where too high mercury levels have been detected in regularly consumed fish species [28]. Moreover, the blood lead levels above the reference may be attributed to the consumption of agricultural products, such as cassava [32,33,84], and the drinking of contaminated tap water [34], in addition to the ingestion of lead-contaminated (herbal) food supplements [85], exposure to lead-based paints, fuels, and mosquito coils [86,87], and/or the application of lead-containing cosmetics [88]. Whether and which of these causes might be the reason for the levels of these heavy metals in the pregnant Surinamese women is not clear and needs further investigation. This is particularly necessary when considering the relatively high frequency of preterm births found in the current study (12.5%) as well as in a previous study that was also conducted in Suriname [44].

Nevertheless, that the high number of blood levels above the reference levels of these compounds was apparently not associated with adverse birth outcomes in the current study may be tentatively explained by the relatively small number of participants included in the study. The study was initially designed and powered for 1200 participants based on the effect size for the longitudinal study with this number of women. However, as mentioned before, at the time of the current study, blood specimens’ data were only available for 400 women. Consequently, the effect size was smaller than the effect size for which the study was originally powered. Obviously, the resulting lower power is a limitation of the current study. The remaining blood specimen data are anticipated to be available in the near future.

In addition, the high frequency of women with blood metal levels above public health action reference levels may not lead to immediate adverse health effects in pregnant women and newborns, but rather manifest as neurodevelopmental disorders in children many years after birth [89,90,91], childhood overweight or obesity [42], impaired liver or kidney functions [92], lower bone density [92,93] or cardiovascular disorders at a more advanced age [94,95,96].

The association of each of the studied covariates in the current study with adverse birth outcomes has been well documented [55,58,97,98,99,100,101,102]. In accordance with these data, we found statistically significant risks for the occurrence of preterm birth and low birth weight in women suffering from hypertension or diabetes mellitus. Why no association was found between other risk factors for adverse birth effects, such as underlying anemia in the mother, maternal age over 35 years, multiparity, and pre-pregnancy smoking [58,102], with any of the negative outcomes is not clear but may due to the limited sample size [103].

There is also no explanation at hand for the apparent risk reduction in preterm birth in women who had used alcohol before their pregnancy and who were at the same time suffering from diabetes mellitus or had used prescription medicines, such as anti-anemic drugs. However, some studies have shown an independent relationship of diabetes mellitus and preterm birth in pregnant women [104,105], which emphasizes the importance of screening pre-pregnancy and during pregnancy for diabetes mellitus as a public heath intervention [106].

The apparent attenuation of the risk of preterm birth in women who had been exposed to higher lead concentrations and had also used prescription medicines may tentatively be attributed to interactions of both substances and lead to changes in their pharmacological fate and modification of the risk for this adverse birth outcome. This may hold true for, for instance, iron supplements, which are often prescribed for anemia in pregnant Surinamese women [107]. This can be explained by the fact that several heavy metals, including lead, share the same transporter proteins and receptors with iron, [108,109] causing anemia [92,110], and thus modifying the risk of preterm birth [111]. Obviously, both this supposition and that mentioned in the preceding paragraph must be verified in future studies.

In summary, the results of the current study suggest that there were no associations between prenatal exposure to mercury, manganese, or lead and adverse birth outcomes. However, the small sample size of the study decreases its statistical power, warranting caution when trying to extrapolate these findings to the general population. For the same reason, the absence of an association between the high mercury, manganese, or lead blood levels and adverse birth outcomes must be considered with care. The same holds true for the confounding effects of underlying diabetes mellitus and/or hypertension, as well as the use of prescription medicines and alcohol use.

Nevertheless, our data warrant more careful assessments of pregnant women for risk factors for adverse birth outcomes, such as prenatal exposure to hazardous environmental compounds; underlying (chronic) diseases, such as diabetes mellitus; the use of certain prescription medicines; and pre-pregnancy alcohol use, not only separately but also when these risks are present in various combinations. As shown by the current data, these practices will likely help improve environmental and health policies to ensure the safety of (pregnant) women and their newborns in Suriname. Future studies should include an increased number of pregnant women and specifically enroll women with underlying anemia, diabetes mellitus, and/or hypertension, as well women using prescription medicines, such as anti-anemic drugs in order to eliminate type II errors.

## 5. Conclusions

The findings of this study suggest that there were no statistically significant relationships between mercury, manganese, and lead blood levels above reference (high) values and adverse birth outcomes, including stillbirths, preterm birth, low birth weight, and low Apgar score. These findings and the alarming frequency of blood levels of mercury, manganese, and lead above the reference levels, indicate an urgent need for environmental health research and public health policy interventions to evaluate and protect pregnant Surinamese women more meticulously for potential risk factors for adverse birth outcomes and the effects in the long term for mothers and their newborns.

## Figures and Tables

**Table 1:** **General characteristics of the study population (N = 380)**.

	Number *	Proportion
Total number of participants	380	100.0%
**Age categories**		
16–19 years	38	10.0%
20–34 years	284	74.7%
35+ years	58	15.3%
**Region of residence**		
Urban–coastal	251	66.1%
Rural–coastal	85	22.4%
Rural–interior	44	11.6%
**Household income**		
<USD 75	112	31.2%
≥USD 75	247	68.8%
**Education level**		
No or primary education	66	17.5%
Secondary or tertiary education	312	82.5%
**Parity**		
Nulliparous	113	29.9%
Multiparous	265	70.1%
**Food consumption**		
Fish (yes)	364	97.1%
Rice (yes)	362	96.3%
Cassava (yes)	103	27.4%
Plantains (yes)	93	24.7%
Potatoes (yes)	137	36.4%
Bread (yes)	298	98.9%
Leafy vegetables (yes)	373	99.5%
**Blood levels of heavy metals**		
Mercury		
<3.5 µg/L	226	59.5%
≥3.5 µg/L	154	40.5%
Manganese		
<13.0 µg/L	137	36.1%
≥13.0 µg/L	243	63.9%
Lead		
<3.5 µg/dL	299	78.9%
≥3.5 µg/dL	81	21.3%
**Adverse birth outcomes**		
Adverse birth outcomes	74	19.5%
Stillbirths	17	4.5%
Gestational age at birth < 37.0 weeks	50	13.3%
Birth weight < 2500 g	43	11.6%
Apgar scores < 7 at 5 min	19	5.2%
**Covariates**		
Blood glucose level ≥ 6.1 mmol/L (elevated)	15	10.9%
Blood pressure > 120/80 mm Hg (elevated and high)	55	32.7%
Anemia < 6.8 mmol/L (severe, moderate, and mild)	203	84.6%
Prescription medicine use (yes)	224	58.9%
Alcohol use 3 months before pregnancy (yes)	173	57.8%
Smoking 3 months before pregnancy (yes)	18	4.8%

* Numbers may not add up to 380 due to missing values: preterm birth (n = 2); low birth weight (n = 5); low Apgar score (n = 12); household income (n = 21); educational level (n = 2); food consumption: fish (n = 5); rice, cassava, plantains, potatoes, bread, leafy vegetables (n = 4); blood glucose levels (n = 242); blood pressure levels (n = 212); anemia (n = 140); parity (n = 2); alcohol use 3 months before pregnancy (n = 18); smoking 3 months before pregnancy (n = 4).

**Table 2:** Association of blood metal levels and covariates with each of the adverse birth outcomes.

Variables	Live Birth Status			Preterm Birth < 37.0 Weeks			Birth Weight < 2500 g			Apgar Score < 7 at 5 min		
	Number of Women		χ^2^- Test Result	Number of Women		χ^2^- Test Result	Number of Women		χ^2^- Test Result	Number of Women		χ^2^- Test Result
	Stillbirth	Live Birth		<37 Weeks	≥37 Weeks		<2500 g	≥2500 g		Yes	No	
Mercury												
<3.5 µg/L	7	219	2.472, *p* = 0.134	32	193	0.254, *p* = 0.648	31	194	2.270 *p* = 0.144	9	211	1.284, *p* = 0.337
≥3.5 µg/L	10	144		19	134		13	137		10	138	
Manganese												
<13.0 µg/L	9	128	2.402, *p* = 0.195	13	122	2.684, *p* = 0.117	13	120	0,764, *p* = 0.408	9	125	1.038, *p* = 0.333
≥13.0 µg/L	8	235		38	205		31	211		10	224	
Lead												
<3.5 µg/dL	11	288	2.073, *p* = 0.220	35	263	**3.682,** ***p* = 0.065**	32	262	0.974, *p* = 0.333	13	275	1.140, *p* = 0.266
≥3.5 µg/dL	6	75		16	64		12	69		6	74	
Age categories												
16-19 years	1	37	0.375, *p* = 0.918	5	33	0.096, *p* = 1.000	7	31	1.938, *p* = 0.358	2	34	0.015, *p* = 1.000
20-34 years	13	271		39	244		30	250		14	261	
35+ years	3	55		7	50		7	50		3	54	
Blood glucose levels												
<6.1 mmol/L	7	116	1.281, *p* = 0.254	14	108	**6.139,** ***p* = 0.028**	16	106	0.001, *p* = 1.000	7	113	1.696, *p* = 0.214
≥6.1 mmol/L	2	13		5	9		2	13		2	11	
Blood pressure levels												
≤120/80 mm Hg	5	108	2.542, *p* = 0.180	14	98	1.764, *p* = 0.246	10	102	**6.719,** ***p* = 0.015**	6	103	3.146. *p* = 0.117
>120/80 mm Hg	6	49		11	43		13	42		7	44	
Anemia												
No anemia ≥ 6.8 mmol/L	2	35	0.000, *p* = 1.000	3	33	2.178, *p* = 0.224	2	34	1.913, *p* = 0.273	2	32	0.027 *p* = 1.000
Anemia < 6.8 mmol/L	11	192		37	165		28	174		13	183	
Previous live births												
Nulliparous	5	108	0.002, *p* = 1.000	10	103	3.062, *p* = 0.100	13	99	0.006, *p* = 1.000	6	103	0.031, *p* = 0.803
Multiparous	12	253		41	222		31	230		13	244	
Prescription medicine use												
Yes	11	213	0.244, *p* = 0.802	39	184	**7.443,** ***p* = 0.006**	29	195	0.790, *p* = 0.416	13	202	0.824, *p* = 0.476
No	6	150		12	143		15	136		6	147	
Alcohol use 3 months before pregnancy												
Yes	5	168	2.415, *p* = 0.141	17	155	**4.969,** ***p* = 0.034**	13	158	**6.114,** ***p* = 0.015**	6	161	2.097, *p* = 0.164
No	12	177		34	154		30	156		13	170	
Smoking 3 months before pregnancy												
Yes	0	18	0.895, *p* = 1.000	1	17	1.049, *p* = 0.487	0	18	2.546, *p* = 0.146	1	17	0.004, *p* = 1.000
No	17	341		50	306		44	309		18	328	

Fisher’s exact test, *p* < 0.10 is used as cut-off for inclusion in (multivariate) logistic regression models.

**Table 3:** Crude and adjusted odds ratios with 95% confidence interval for preterm birth.

Logistic Regression Model Analysis for Preterm Birth				
	Crude Model		Adjusted Model	
Variable	Odds Ratio (95% CI)	*p* Value	Odds Ratio (95% CI)	*p* Value
Blood lead level				
<3.5 µg/dL	reference		reference	
≥3.5 µg/dL	1.88 (0.98–3.60)	0.058	1.38 (0.40–4.79)	0.615
Blood glucose level				
<6.1 mmol/L	reference		reference	
≥6.1 mmol/L	4.29 (1.26–14.62)	**0.020**	5.58 (1.38–22.53)	**0.016**
Prescribed medication				
No	reference		reference	
Yes	2.53 (1.28–5.00)	**0.008**	2.1 (0.43–10.16)	0.358
Alcohol use 3 months before pregnancy				
No	reference			
Yes	0.50 (0.27–0.93)	**0.028**	0.36 (0.11–1.145)	0.083

**Table 4:** Crude and adjusted odds ratio with 95% confidence interval (CI) for low birth weight.

Logistic Regression Model Analysis for Low Birth Weight				
	Crude Model		Adjusted Model	
Variable	Odds Ratio (95% CI)	*p* Value	Odds Ratio (95% CI)	*p* Value
Blood pressure levels				
≤120/80 mm Hg	reference		reference	
>120/80 mm Hg	3.16 (1.28–7.76)	**0.012**	2.72 (1.081–6.86)	**0.034**
Alcohol use 3 months before pregnancy				
No	reference		reference	
Yes	0.43 (0.22–0.85)	**0.015**	0.47 (0.18–1.23)	0.124

## Data Availability

Not applicable.
